# Anxiogenic Effects of Developmental Bisphenol A Exposure Are Associated with Gene Expression Changes in the Juvenile Rat Amygdala and Mitigated by Soy

**DOI:** 10.1371/journal.pone.0043890

**Published:** 2012-09-05

**Authors:** Heather B. Patisaul, Alana W. Sullivan, Meghan E. Radford, Deena M. Walker, Heather B. Adewale, Bozena Winnik, Janis L. Coughlin, Brian Buckley, Andrea C. Gore

**Affiliations:** 1 Department of Biology, North Carolina State University, Raleigh, North Carolina, United States of America; 2 Keck Center for Behavioral Sciences, North Carolina State University, Raleigh, North Carolina, United States of America; 3 Division of Pharmacology and Toxicology, Institute for Neurosciences, and Institute for Cellular and Molecular Biology, University of Texas at Austin, Austin, Texas, United States of America; 4 Joint Graduate Program of Toxicology, a Joint Institute of Rutgers University and the University of Medicine and Dentistry of New Jersey, Piscataway, New Jersey, United States of America; 5 Environmental and Occupational Health Science Institute, Piscataway, New Jersey, United States of America; University of Medicine & Dentistry of NJ - New Jersey Medical School, United States of America

## Abstract

Early life exposure to Bisphenol A (BPA), a component of polycarbonate plastics and epoxy resins, alters sociosexual behavior in numerous species including humans. The present study focused on the ontogeny of these behavioral effects beginning in adolescence and assessed the underlying molecular changes in the amygdala. We also explored the mitigating potential of a soy-rich diet on these endpoints. Wistar rats were exposed to BPA via drinking water (1 mg/L) from gestation through puberty, and reared on a soy-based or soy-free diet. A group exposed to ethinyl estradiol (50 µg/L) and a soy-free diet was used as a positive estrogenic control. Animals were tested as juveniles or adults for anxiety-like and exploratory behavior. Assessment of serum BPA and genistein (GEN), a soy phytoestrogen, confirmed that internal dose was within a human-relevant range. BPA induced anxiogenic behavior in juveniles and loss of sexual dimorphisms in adult exploratory behavior, but only in the animals reared on the soy-free diet. Expression analysis revealed a suite of genes, including a subset known to mediate sociosexual behavior, associated with BPA-induced juvenile anxiety. Notably, expression of estrogen receptor beta (*Esr2*) and two melanocortin receptors (*Mc3r*, *Mc4r*) were downregulated. Collectively, these results show that behavioral impacts of BPA can manifest during adolescence, but wane in adulthood, and may be mitigated by diet. These data also reveal that, because ERβ and melanocortin receptors are crucial to their function, oxytocin/vasopressin signaling pathways, which have previously been linked to human affective disorders, may underlie these behavioral outcomes.

## Introduction

In species from rodents to humans, developmental exposure to endocrine disrupting compounds (EDCs) affects the emergence of sexually dimorphic behaviors sensitive to the organizational effects of steroid hormones [1]. Perinatal exposure to the plastics component Bisphenol A (BPA) alters sociosexual and anxiety-related behaviors in rodents (reviewed in [2]) and non-human primates [3], in age- and sex-specific manners. In nearly all of these prior studies, behavior was assessed in adulthood, and the specific mechanisms by which BPA altered behavior were largely unexplored. Emerging studies have associated prenatal BPA exposure with elevated hyperactivity and anxiety in young girls [4], [5] suggesting the possibility that developmental exposure to BPA and other EDCs might contribute to the growing prevalence of behavioral- and mood-related disorders in children [6], [7]. What remains to be established, and was addressed by the present studies, are (1) how chronic, low dose oral exposure to BPA throughout development impacts affective behavior during adolescence and adulthood; (2) the underlying molecular changes to the nervous system related to these behavioral effects, and (3) the potential for dietary intervention to mitigate BPA effects.

BPA is used in numerous household products including polycarbonate plastic food containers, the epoxy linings of canned foods, and thermal paper receipts [8], [9], [10]. BPA exposure is nearly ubiquitous in the United States [11] and occurs primarily via consumption of food and beverages from which BPA has leached from the container. BPA is hypothesized to act, primarily, by interacting with estrogen receptors [12], but we have previously shown that the anxiogenic properties of BPA exposure are not consistently recapitulated by estrogen exposure [13]. Other studies demonstrate BPA effects on the thyroid hormone receptor [14] and dopamine signaling pathways [15], indicating that the mechanism of action is more complex. Additionally, work with the viable agouti mouse (Avy) has revealed that the soy phytoestrogen genistein (GEN) can counter the hypomethylating properties of BPA resulting in a coat color shift, suggesting epigenetic change as an alternative mechanism of action [16].

GEN and other isoflavone phytoestrogens are estrogen-like compounds produced by plants, most notably soy and other legumes. For decades, they have been recognized to interact with the mammalian endocrine system [17], [18], [19]. Like BPA and other synthetic EDCs, they have historically been thought to act primarily through estrogen receptors [20], but they are also tyrosine kinase inhibitors [21], [22], and modulate DNA methylation and chromatin configuration [23]. The observation that GEN can mitigate effects of BPA on DNA methylation [16] suggests that the mechanisms of action of these compounds are not only distinct but can also act in opposition. For example, the impact of BPA on oocyte meiosis depends, at least in part, on diet [24], further emphasizing the interactive potential of anthropogenic and naturally-occurring EDCs.

Although marketed as a therapeutic to alleviate the physiological and mood changes associated with menopause, the experimental evidence for soy phytoestrogen impacts on sociosexual behavior is inconsistent and appears to vary depending on exposure period and sex [25], [26], [27]. While some studies have shown that life-long soy intake can result in anxiolytic effects in rodents [28], [29], others have shown that adult-only intake can be anxiogenic in males yet anxiolytic in females when endogenous estrogen levels are high and exploratory drive is naturally heightened [30].

The current study addressed several important gaps identified above. First, effects of BPA and soy phytoestrogens were tested on sexually dimorphic anxiety-related behaviors as juveniles and adults. This approach enabled us to distinguish the importance of developmental age at testing, and to explore interactions between BPA and phytoestrogens. To gain further insight into what molecular changes might underlie the behavioral phenotype, we used a low-density PCR array to identify transcriptional changes within the amygdala (AMYG), chosen because of its fundamental role in the processing of sociability, fear, anxiety and other emotional responses [31]. The 48 genes selected for analysis in this study were chosen because they are known to influence sociosexual behavior, are modified by estrogen, or are vulnerable to BPA exposure. To ensure that exposure to BPA and the phytoestrogens were in a range considered relevant to humans, internal dose of BPA and GEN were assessed throughout the dosing period.

## Methods

### Ethics Statement

Animal care, maintenance, and surgery were conducted in accordance with the applicable portions of the Animal Welfare Act and the U.S. Department of Health and Human Services “Guide for the Care and use of Laboratory Animals” and were approved by the North Carolina State University (NCSU) Institutional Animal Care and Use Committee (IACUC). All procedures were approved and monitored by the supervising veterinarian for the duration of the project.

### Animal Care and Exposure

Wistar rats bred in house and reared on a phytoestrogen-free diet over several generations were used for these experiments. Due to the large number of animals needed and limited space available for breeding, matings were split into 4 cohorts, all bred approximately one month apart. All dams were housed individually in two humidity-and-temperature controlled rooms (segregated by diet), each with a 14 h∶10 h light, dark cycle (lights on from 0200 to 1400 h) at 23°C and 50% average relative humidity at the Biological Resource Facility at NCSU.

Exposure began on gestational day (GD) 6 and continued until postnatal day (PND) 40 (GD 0 defined as day of sperm plug detection; PND 0 defined as day of birth) so that exposure covered post-implantation gestation through peri-puberty. On PND 21 pups were weaned into same sex and exposure groups (3–5 pups per cage), and ear punched for identification. At 3 months of age they were separated into same-sex pairs. There were a total of 5 exposure groups: **Soy** (soy diet); **BPA + Soy** (soy diet plus water containing BPA); **Soy-free** (soy-free diet); **BPA** (soy-free diet plus water containing BPA) and **EE** (soy-free diet plus water containing EE). The soy-based diet was Purina 5001 [32] and the soy-free diet was Teklad 2020 (Harlan). Phytoestrogen levels in the Purina 5001 diet [33], [34] approximate levels in a traditional Asian diet or consumed by infants reared on soy formula [26]. The pharmacokinetics of GEN and other phytoestrogens delivered by this diet have been well characterized in the rat [34], [35]. Dams were placed on their respective diet at least a week prior to mating and remained on their assigned diet for the duration of the experiment. BPA ((2,2-bis(4-hydroxyphenyl)propane; CAS No. 80-05-7; Lot 11909; USEPA/NIEHS standard provided to HBP); 1 mg/L of water) and the positive control ethinyl estradiol (EE; 50 µg/L) were administered via drinking water. This dose of BPA was chosen based on prior studies utilizing this method of exposure [36], [37], [38] to produce serum levels in the human range. The scale of the study precluded us from examining more than one dose of BPA. Each water solution (BPA and EE) was prepared initially as a 10× stock solution. Working solutions were prepared by adding 50 ml of stock solution to 450 ml filtered water (500 ml per bottle total). Cohort 1 EE dams drank less than controls or dams consuming BPA. To encourage them to drink, for cohorts 2 and 3, 1 g of sucrose was added to the 10× EE stock solution to increase palatability.

Water consumption for each cage was measured bi-weekly by weighing the bottles empty, full (500 ml) and then after a few days to assess intake. For the pups, estimated intake per pup was estimated by dividing consumption by the number of pups in the cage. Daily intake was used to estimate daily ingestion of BPA or EE (Tables 1 and 2).

**Table 1:** Mean Dam Fluid Intake (ml) and Exposure Levels (µg) of BPA or EE.

Dam Exposure Group	Mean Daily Gestational Intake (ml)	Mean Daily Gestational Exposure to BPA or EE (µg)	Mean Daily Lactational Intake (ml)	Mean Daily Lactational Exposure to BPA or EE (µg)
Soy-free	60.6±1.7	0	57.9±2.4	0
Soy	58.7±2.3	0	86.1±17.3	0
BPA	35.2±2.2	35.2±2.2	71.8±18.5	71.8±18.5
BPA + Soy	55.6+4.5	55.6±4.5	105.6±5.7	105.6±5.7
EE	21.0±4.9	1.1±0.3	28.8±5.1	1.4±0.3

Average daily water consumption was quantified from the dams during mid-gestation and mid-lactation, from which average daily BPA or EE was calculated. Soy exposure occurred via diet only and therefore is not included.

**Table 2:** Mean Pup Intake (ml) and Exposure Levels (µg) of BPA or EE.

Pup Exposure Group	Sex	Mean Daily Intake (ml)	Mean Daily Exposure to BPA or EE (µg)
Soy-free	Female	11.9±0.1	0
	Male	11.8±2.0	0
Soy	Female	18.7±2.8	0
	Male	16.8±1.7	0
BPA	Female	22.4±2.8	22.4±2.8
	Male	18.2±2.0	18.2±2.0
BPA + Soy	Female	23.2±1.7	23.2±1.7
	Male	24.8±2.1	24.8±2.1
EE	Female	16.9±1.8	0.9±0.1
	Male	18.5±5.1	0.9±0.3

Average daily water consumption was quantified from the pups during postnatal days 21–40, from which average daily BPA or EE was calculated. Soy exposure occurred via diet only and therefore is not included.

### Serum Analysis of BPA and GEN

BPA, GEN and the glucuronide metabolite of GEN were isolated and identified in the dams and PND 12 animals as described previously [39] (See Supporting Information S1). An attempt was made to quantify levels in a subset of juveniles but those results could not be reported with confidence.

### Behavior

Juvenile behavior was assessed after weaning but prior to puberty (PNDs 24–28) using the light/dark box (L/D box) and elevated plus maze (EPM) as previously described [40]. Adult behavior was assessed from PNDs 60–70 using the EPM. Because it is well established that female exploratory behavior is cycle-dependent [30], [41], all females were tested in estrus. Cycle phase was identified by vaginal lavage [42]. All behavior was video recorded and subsequently scored by an observer, blind to the treatment groups, using Stopwatch (courtesy of the Center for Behavioral Neuroscience, Emory University, Atlanta, GA, USA).

### Gene Expression Analysis

A subset of animals tested for juvenile behavior was sacrificed on PND 34, and the brains removed and flash frozen on powdered dry ice. Using a cryostat, each brain was sectioned to reveal the caudal border of the amygdala was visible (identified with the assistance of a brain atlas (Paxinos and Watson plates 49–58), removed via micropunch, and shipped to Dr. Gore at University of Texas at Austin for further analysis according to their established protocols [43], [44] (See Supporting Information S1). Relative expression was determined using the comparative Ct method [45], [46], [47], with each sample normalized to *Gapdh*, and data calibrated to the median **Δ**Ct for the group with the lowest expression set at 1.0 [44]) to determine fold change in expression for each sample.

### Data Analysis

Because the EE group was included to specifically test the hypothesis that any observed BPA effects were estrogenic, it was not incorporated in the overall analysis, but rather compared to the group of interest by a t-test, when appropriate. Behavior reported as a percent was analyzed by logistic regression. For the remaining behavioral and gene expression endpoints, three-way ANOVA with gender, BPA exposure, and diet as factors, was used to identify main effects and interactions. No effect of gender was found for juvenile behavior so the data were collapsed across gender for all subsequent analyses. Group differences between groups maintained on the same diet were then identified using post-hoc two-sample separate variance t-tests. A similar approach was used to analyze behavioral endpoints in the adults, but the analysis was conducted within sex.

For gene expression data, relative expression was analyzed by three-way ANOVA with gender, BPA exposure and diet as factors to determine main effects and interactions. Data were first tested for outliers using the z-score of the residuals from the initial regression and was considered an outlier if it was greater than 2.5 standard deviations from the initial line of best fit. Confirmed outliers were excluded from final analysis. If expression data did not meet the assumptions for ANOVA, the data were transformed (ln) and reanalyzed. In a few cases, transformed data did not meet the assumptions for parametric testing. In those cases, non-parametric tests were used to determine differences between the groups (See Table S1). Because interactions of variables cannot be determined using non-parametric testing, data were coded according to each variable or combination of variables and analyzed using a Kruskal-Wallis or Mann-Whiney U test. To identify group differences within sex for the post-hoc analysis, two-sample t-tests were performed with separate variance.

## Results

### Litter Data

Average litter size (*P* = 0.381), and sex ratio within the litters (*P* = 0.886) did not significantly differ across treatment groups.

### Exposure and Internal dose of BPA and GEN

Dam water consumption was subdivided into amounts consumed during mid-gestation and mid-lactation (Table 1). One-way ANOVA indicated a significant effect of exposure group (*P*≤0.001). Subsequent analysis with a Fisher's LSD post-hoc test revealed that during gestation, BPA exposed dams drank significantly less water than all other groups (*P*≤0.001), and EE exposed females drank significantly less across gestation (*P*<0.001) and lactation (*P*≤0.003) compared to controls. Importantly however, intake in these groups was sufficiently high enough to ensure that dehydration was not a potential confound. Pup intake over PNDs 21–40 was estimated for each sex (Table 2) and analyzed in a two-way ANOVA with gender and exposure as factors. A significant exposure effect was identified (*P*≤0.004) with BPA-exposed pups of both sexes (regardless of diet) drinking significantly more water than unexposed groups (*P*≤0.05; Table 2). An estimate of daily BPA and EE intake was then calculated for the dams and pups from these water intake levels (Tables 1 and 2).

Internal dose was established by quantifying free BPA and GEN, as well as conjugated GEN plasma levels in a subset of PND 12 and all dams on the day of weaning (Table S1 A–B). Samples from the pups were pooled to obtain sufficient volume for assessment. For all groups, free BPA levels were less than 2 ng/ml; a range that approximates the current estimated mean serum levels in humans [48]. Levels in the pups were lower than in the dams, a result which is consistent with prior work indicating that it does not lactationally transfer efficiently [49]. Dam GEN levels were in the range of vegetarians and other populations that regularly consume soy-rich foods (Table S1), but well below those seen in soy-formula fed infants [26]. GEN levels were lowest in the PND 12 pups, reflecting poor lactational transfer [49], [50]. An attempt was made to quantify serum levels in the juveniles but those results could not be reported with confidence because the values were near the limit of detection and other technical issues. Trace levels of free BPA were found in some of the unexposed controls a result which may reflect contamination (from the collection materials or diet), or an artifact since the levels were close to the limit of detection (0.1 ng/ml).

### Juvenile Exploratory and Anxiety-like Behavior

**Figure 1:**
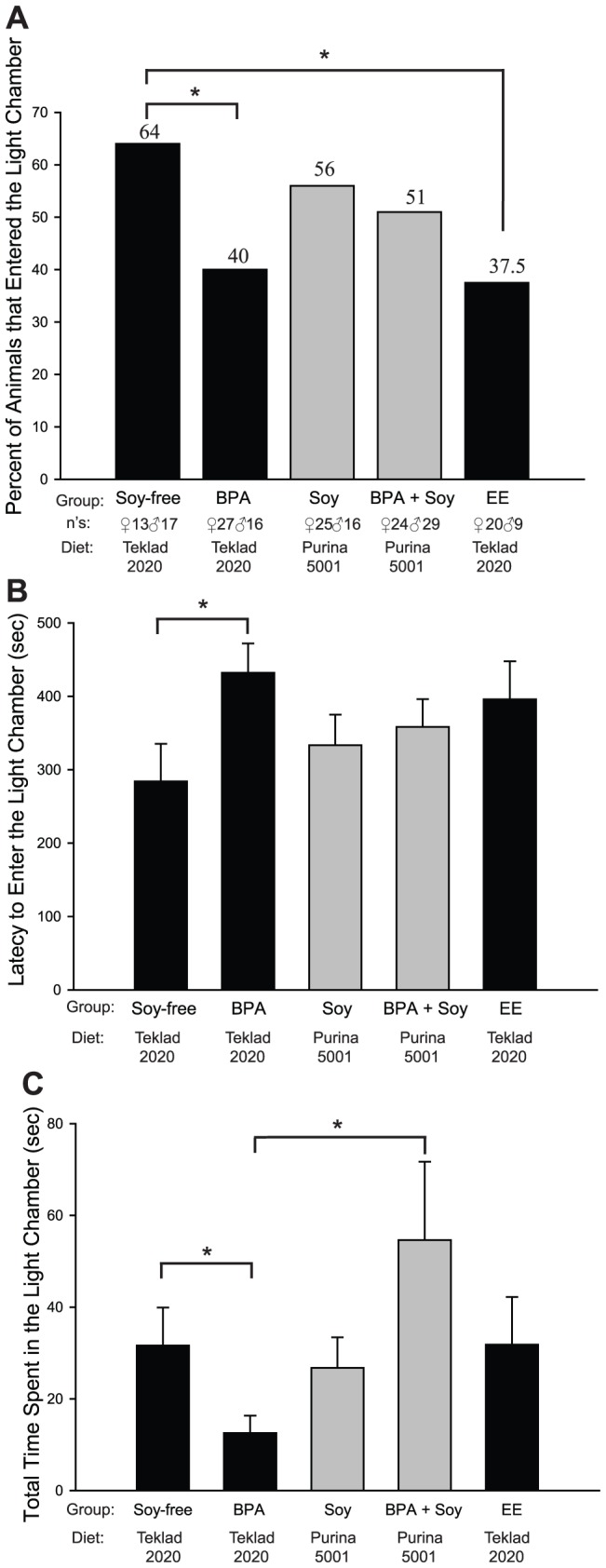
BPA, EE and dietary effects on juvenile Wistar rat Light/Dark Box activity. No significant effect of gender was identified, therefore the data were collapsed across sexes. Animals maintained on the soy-based diet are depicted in gray and animals maintained on the soy-free diet are depicted in black. (A) BPA and EE exposure significantly decreased the percent of animals entering the lit chamber compared to controls on the same diet (Soy-free). (B) BPA exposure significantly increased latency to enter the lit chamber compared to the Soy-free controls. (C) Animals exposed to BPA and maintained on the soy-free diet spent significantly less time in the lit chamber than the Soy-free controls, and also the BPA-exposed animals maintained on the soy-based diet (BPA + Soy). Graphs depict mean ± SEM, *P≤0.05.

Juvenile behavior was first assessed in the Light/Dark box, an apparatus that appraises an animal's motivation to leave the reassuring confines of a darkened enclosure to explore an aversive, brightly lit chamber [51]. No significant effect of sex was found for any of the assessments, nor any significant interactions with sex; so, for subsequent analyses, the data were collapsed across gender. Among the animals maintained on a soy-free diet, the percent of BPA and EE exposed animals entering the lit chamber was significantly lower than for the control animals (*P*≤0.05; Figure 1A) indicating a higher level of anxiety and reluctance to explore the novel environment in the treatment groups. No effect of BPA was found among the soy-fed animals. A significant effect of BPA exposure was found on latency to enter the lit chamber (Figure 1B), but only among animals maintained on the soy-free diet (*P*≤0.05) with BPA exposed animals taking longer to enter the lit chamber. A diet by BPA exposure interaction (*P*≤0.05) was identified for the overall amount of time spent in the lit chamber, with BPA exposed animals on the soy-free diet spending significantly less time in the lit chamber than control animals on the same diet (Figure 1C; *P≤*0.05). Among the animals maintained on the soy-based diet, however, BPA exposure elevated the time spent in the lit chamber indicating a significant modifying effect of diet. Collectively, these results show that developmental low dose BPA exposure induces anxiogenic responses and that concurrent consumption of a soy-based diet is capable of counteracting these effects. EE exposure did not recapitulate the behavioral effects of BPA exposure, suggesting that the mechanism is not classically estrogenic.

**Figure 2:**
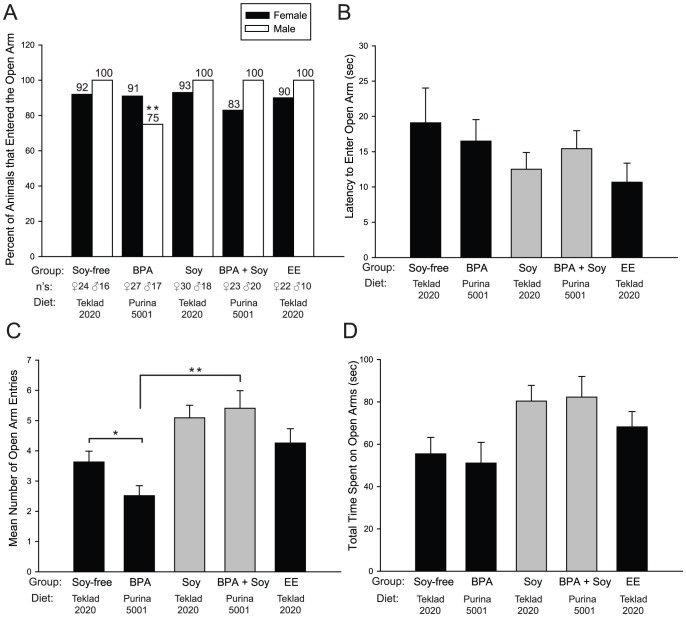
BPA, EE and dietary effects on juvenile Elevated Plus Maze (EPM) activity. (A) BPA exposure significantly decreased the percent of males that entered an open arm. No significant effect of gender was found for any subsequent measure, therefore the data were collapsed across sexes. (B) There were no significant group differences in latency to enter the open arms. (C) A main effect of diet on mean number of open arm entries was identified (not depicted), with soy-fed animals making significantly more entries. BPA exposed animals on the soy-free diet (BPA) made fewer entries compared to the diet matched controls (Soy-free) and also the BPA exposed animals on the soy diet (BPA + Soy). (D) Soy-based diet significantly increased time spent on the open arms, regardless of BPA exposure. EE had no significant effect on any EPM endpoint examined. Graphs depict mean ± SEM, *P≤0.05, **P≤0.001, # main effect of diet; *P*≤0.05.

The animals were then subjected to the elevated plus maze (EPM) as an additional assessment of anxiety. This maze has two arms with high protective walls, and two arms with no walls. This apparatus assesses the animal's reluctance to leave the relative safety of the walled arms to explore the more aversive, open arms [52]. Activity in the closed arms is not mood dependent and thus regarded as a marker of general activity rather than anxiety (See Figure S1). The percent of BPA exposed males entering the open arms was significantly lower than all other groups (*P*≤0.001; Figure 2A) with only 75% of males entering the open arms. For the remaining measures, the data were collapsed across gender because no main effect of sex was identified. Latency to enter the open arms was not significantly different between groups (Figure 2B). Two-way ANOVA revealed a main effect of diet (*P*≤0.002) on number of open arm entries, with the soy-fed animals making more entries than those maintained on soy-free diet regardless of BPA exposure (Figure 2C). Follow-up t-tests revealed that BPA exposed animals on the soy-free diet made significantly fewer open arm entries than control animals on the same diet (*P*≤0.05), while EE exposure had no effect. Of the animals exposed to BPA, those maintained on the soy-based diet made significantly more entries (*P*≤0.001) indicating a protective effect of soy diet. Two-way ANOVA also revealed a significant main effect of diet on time spent on the open arms (*P*≤0.001; Figure 2D) with animals on the soy-free diet showing less activity on the open arms than those maintained on the soy-based diet. BPA had no effect on this measure, regardless of diet. These data are consistent with those obtained from the Light/Dark box in that they reveal the anxiogenic effects of BPA exposure, and the capacity of a soy-based diet to mitigate this behavioral change.

### Gene Expression Changes in the Juvenile Amygdala Associated with Disrupted Behavior

**Figure 3:**
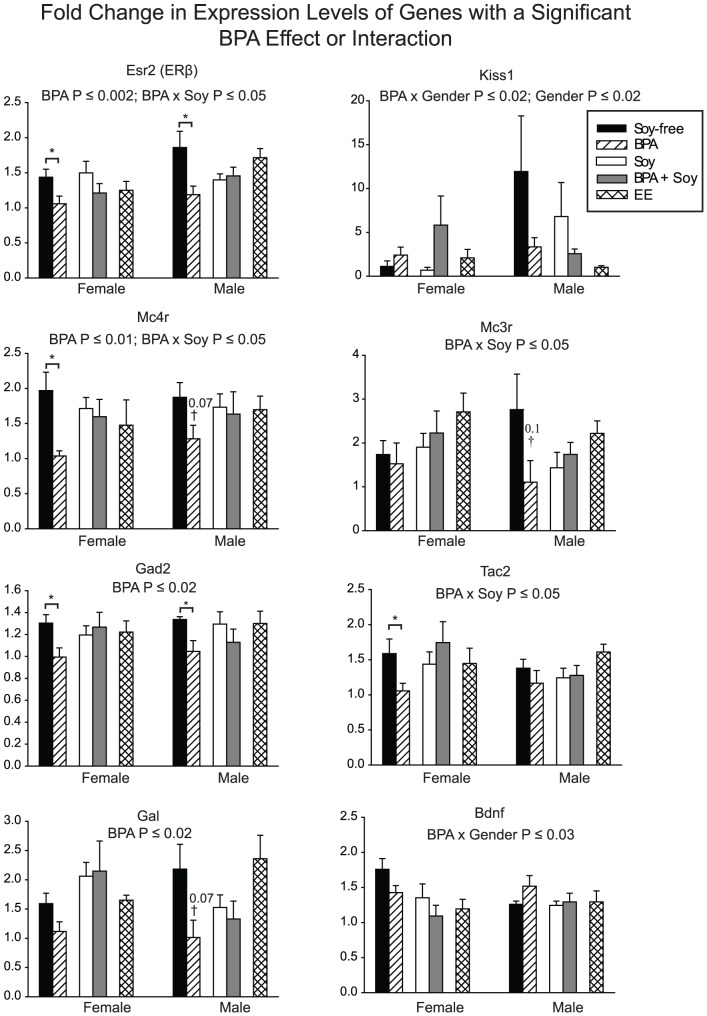
Fold change in juvenile amygdalar gene expression levels. Of the 48 genes examined, a significant main effect of, or interaction with, BPA was found for the 8 genes shown here. The genes depicted met the threshold criteria established for biological significance. For each gene, the raw *P*-values obtained for the main and interaction effects of BPA are indicated. Significant group differences within each sex, identified by post-hoc t-tests, are also shown. *Esr2* (ERβ) and *Gad2* expression were significantly down-regulated by BPA in both sexes compared to Soy-free controls. *Tac2* and *Mc4r* were significantly down-regulated by BPA exposure in females. Among the soy-fed animals, no significant effect of BPA was identified for any gene, indicating a protective influence of soy. Graphs depict mean fold change in expression levels ± SEM. *P≤0.05, †*P*≤0.1 compared to soy-free controls.

A subset of the juveniles was sacrificed on PND 34 to identify gene expression levels in the amygdala associated with BPA-induced behavioral change. Of the 48 genes selected for analysis, a main effect of BPA exposure and/or an interaction with BPA was found for 13 genes when compared by 3-way ANOVA (with factors of sex, BPA and soy; see Table S2). No effects survived a false-discovery rate correction [53], [54]. Two housekeeping genes were included on the array (*18s* and *Gapdh*). Both displayed a small but significant effect of diet on relative expression (∼20% when collapsed across all other variables). Thus, in order to maintain consistency with previous publications [44], the data were normalized to *Gapdh* and, as a conservative approach, only genes displaying greater than 20% change in expression were considered in the subsequent analysis to identify expression changes associated with BPA and/or soy intake. This approach identified 8 genes from the list of 13 (Figure 3).

Generally, BPA exposure decreased expression by about 1.5-fold. Four genes *(Esr2 (ERβ)*, *Mc4r*, *Mc3r*, *Tac2*) exhibited a BPA by diet interaction (*P*≤0.05), with soy diet mitigating the effect of BPA, and all four decreased ∼1.5–2 fold with BPA exposure alone. These candidate 8 genes were then analyzed within sex using a two-sample t-test. *Bdnf* and *Kiss1* expression were affected by BPA exposure in a sex specific manner. Both genes were sexually dimorphic with *Kiss1* being ∼9-fold greater in males than females and *Bdnf* expression being ∼1.5-fold lower in males when compared to females. These sex differences were eliminated by BPA exposure. In both sexes, *Esr2* and *Gad2* expression were significantly down-regulated by BPA compared to Soy-free controls (*P*≤0.05). *Tac2* and *Mc4r* (*P*≤0.05) were significantly down-regulated by BPA exposure only in females. Among the soy-fed animals, no significant effect of BPA was identified for any gene, suggesting a protective effect of soy. None of these BPA-associated gene expression changes were recapitulated by EE demonstrating that they do not likely result from strictly estrogenic effects.

### Adult Exploratory and Anxiety-Related Behavior

**Figure 4:**
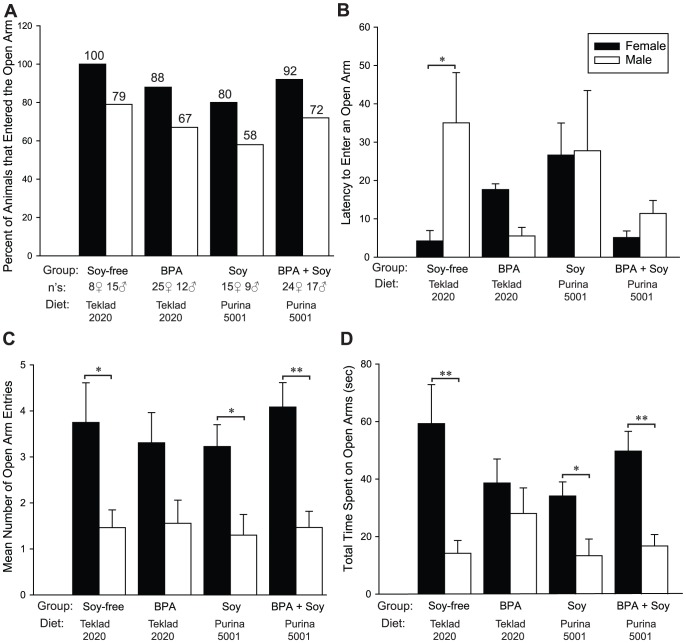
BPA, EE and dietary effects on adult Elevated Plus Maze (EPM) activity. As expected, a significant gender effect was found for all measurements except latency to enter an open arm. In general, BPA exposure eliminated these sex differences. (A) A higher percent of females entered the open arms than males regardless of exposure group. (B) Soy-free males took significantly longer to enter an open arm than females maintained on the same diet. BPA exposure eliminated this sex difference, with a tendency for reversal. Soy diet also eliminated this behavioral sex difference. (C) Females made significantly more open arm entries than males in all groups except those on the soy-free diet and exposed to BPA (BPA). There was no overall effect of BPA or diet on this behavior. (D) Similarly, in all groups except those on the soy-free diet and exposed to BPA, females spent significantly more time on the open arms than males. Graphs depict mean ± SEM, *P≤0.05, ** *P*≤0.001.

Adult exploratory behavior was assessed between PNDs 60–70 in the EPM. EE exposed animals were not included because it produced no significant behavioral effects in the juveniles and all of these animals were sacrificed for inclusion in the gene expression analysis. Adult exploration of both EPM arm types is typically lower than that of juveniles and sexually dimorphic, with females displaying more open arm activity than males [55]. All females were tested on the day of behavioral estrus to control for ovulatory cycle effects, because females are typically most active on the EPM when they are sexually receptive and thus naturally more exploratory [56], [57]. For both sexes, the percent of animals entering the open arms was lowest in the group reared on the soy-based diet, but no significant overall effect of diet, BPA or gender was found for this specific measure (Figure 4A). As expected [57], three-way ANOVA identified a significant effect of sex (*P*≤0.001) on all other EPM activity (Figure 4B-D) with females being more exploratory than males. Because of the strong gender effect, the data were analyzed within sex.

Latency to enter the open arms (Figure 4B) was highly sexually dimorphic, with males taking nearly four times longer to enter the more aversive open arm than females (*P*≤0.05). A significant BPA by diet interaction (*P*≤0.02) was found in females with BPA and soy diet increasing latency when administered independently, but not in combination. In contrast, a significant main effect of BPA exposure (*P*≤0.05) was found in males with BPA feminizing this behavior regardless of diet. Confirmatory t-tests identified a significant sex difference in latency to enter the open arms for only the controls reared on soy-free diet, demonstrating the loss of this sex difference by BPA and soy intake. In general, activity on the open arms was sexually dimorphic, as expected, but this difference was eliminated by BPA exposure in the animals maintained on the soy-free diet, and preserved in the animals consuming the soy-rich diet (Figures 4C and D). Two-way ANOVA (BPA and diet as factors) revealed a significant BPA by diet interaction on the amount of time females spent on open arms (*P*≤0.03), and post-hoc t-tests showed that females spent significantly more time exploring open arms than males, except in the BPA exposed group reared on the soy-free diet (*P*≤0.05; Figure 4D). Sex differences in closed arm activity were also impacted by BPA (see Figure S2). Collectively, these data show that aspects of the anxiogenic action of developmental BPA exposure persist into adulthood, resulting in the loss of behavioral sex differences, but that a soy-rich diet is protective.

## Discussion

The present study reveals that early life exposure to BPA elevates juvenile anxiety in rats of both sexes, but that these effects can be mitigated by concomitant intake of a soy-rich diet. Our data are novel in that they evaluated outcomes of exposure at adolescence, a life stage of great hormonal and neurobiological change, and identified specific gene targets in the amygdala. Furthermore, our data are consistent with recent studies reporting that developmental exposure to BPA is associated with hyperactivity and increased anxiety in young girls [4], [5], but indicate that diet, or other factors, could alleviate these effects.

The behavioral outcomes described here are consistent with prior reports showing that developmental BPA exposure has anxiogenic effects in juvenile and adult C57BL/6J mice [58] and deer mice (*Peromyscus maniculatus*) [59]; and abrogates behavioral sex differences in adult CD-1 mice [60], [61]. A notable difference between the present and prior studies, however, is that here the overall anxiogenic effect of BPA did not strongly persist into adulthood. Several factors could account for this difference, the most obvious of which is a species effect. For example, mice exhibit marked strain differences in anxiety levels [62] and there are numerous important neuroendocrine differences between mice and rats which manifest as species-specific behavioral attributes [63]. Dose is also likely a factor as it varies considerably across studies. Use of more than one dose was beyond the scope of the present study, but future work will be necessary to identify the effective dose range for BPA-related effects on affective behavior. More likely, however is that the minimization of perinatal stress, a well-recognized confound of adult affective behavior, accounts for the less robust effects of BPA on adult anxiety [58], [61], [64]. By using drinking water and diet as the route of administration for BPA and soy phytoestrogens, we did not have to disturb the dams or their pups to dose the animals. Thus, this study is unique because it is the first to orally administer BPA at a dose sufficient to produce human-relevant serum levels [12], [65] while simultaneously minimizing the pronounced confound of perinatal stress. With the adverse impacts of early life stress eliminated, the resulting behavioral changes reported here are more clearly attributable to BPA exposure.

Concomitant administration of a soy-based diet with BPA resulted in attenuation of the BPA effects. In both the Light/Dark box and the EPM, soy largely abrogated the anxiogenic impact of BPA on the percent of rats engaging in the “risky” behavior. Sex differences in adult behavior were maintained except in the case of latency to enter an open arm, where females displayed a longer latency. But again, co-administration of soy and BPA rescued the reversed sex difference induced by BPA exposure. These data are the first to suggest that adverse behavioral outcomes resulting from early life exposure to BPA may be mitigated, at least to some degree, by dietary or other factors. Presumably, this mitigating activity is attributable to GEN, or one (or more) of the numerous other isoflavone phytoestrogens aside from GEN that are commonly found in soy-based foods [16], [26]. Although identification of the specific dietary compounds capable of minimizing the adverse neurobehavioral impacts of BPA and other EDCs is potentially advantageous to pregnant women, it is important to recognize that GEN and other phytoestrogens can also act as EDCs during development. Thus, the critical windows in which they produce effects, and their mechanism(s) of action and interaction on neural systems that mediate behavior must be more clearly elucidated.

The mechanisms by which BPA alters affective behavior remain poorly understood, but the amygdalar gene expression data obtained here identified 8 genes associated with BPA-induced anxiety including *Esr2* (ERβ), *Mc4r*, *Gal*, *Gad2* and *Kiss1*. These genes have previously been associated with sociosexual and affective behaviors. Interestingly, a recent paper showed transgenerational, epigenetic effects of vinclozolin (a fungicide) on the male F3 generation's responses to stress during adolescence [66]. The male F3 vinclozolin-lineage descendants differed from vehicle-lineage counterparts in how adolescent stress affected subsequent performance on tests of anxiety/stress, and that these same animals differed in expression of genes in hippocampus and amygdala, including *Mc3r* and *Mc4r*, similar to results in BPA-exposed F1 rats in the current study.

Notably, *Esr2* and *Mc4r*, changes to which were, in the present study, detected in the amygdala, play crucial roles in the regulation of oxytocin and vasopressin secretion [67], [68], [69]. These two neuropeptides are well-established to be essential for mediating social interactions and affiliation in rodents and primates, including humans [70], [71]. For example, Esr2 agonism has been shown to be anxiolytic [69], [72], and in the paraventricular nucleus (PVN), Esr2 is required to drive estrogen-dependent oxytocin and vasopressin production [73], [74]. α-Melanocyte stimulating hormone (α-MSH), acting through Mc4r, induces dendritic release of oxytocin from magnocellular PVN neurons [68]. Central actions of oxytocin and vasopressin are implicated in a suite of sociosexual behaviors [71], [75] and confer anxiolytic effects [76]. Collectively, these observations suggest that disrupted ontogeny of oxytocin and vasopressin signaling pathways may underlie the observed changes in juvenile affective behavior. In further support of this hypothesis, a recent study using mice found that prenatal BPA exposure had significantly lower whole brain levels of oxytocin and vasopressin just prior to birth compared to unexposed controls [77]. Dysregulation of these neuropeptide signaling pathways has been implicated in a range of childhood affective disorders, including autism [78], [79]. Although oxytocin and vasopressin gene expression levels were not significantly altered by exposure or diet in the present study (Table 2), the amygdala may not be the most salient region to assess expression levels of these neuropeptides because they are generated primarily in the PVN and supraoptic nucleus (SON). Thus, subsequent investigation of oxytocin and vasopressin production in the PVN and SON will be necessary to confirm the hypothesis that these neuropeptides may be altered by early life BPA exposure.

Notably, BPA significantly impacted the expression of *Kiss1*, a gene only recently discovered to be expressed in the amygdala [80]. This gene codes for the peptide kisspeptin, expression of which in the hypothalamus is now recognized to play a critical role in the timing of sexual maturation, female ovulation, and feedback mechanisms on the hypothalamic-pituitary-gonadal (HPG) axis [81]. Kisspeptin neurons are sparse in number and primarily confined to the medial amygdala, with males having more than females. In the present study, this sex difference in amygdalar *Kiss1* expression was also observed, but, unexpectedly, EE did not masculinize expression in females. Instead, it feminized expression in males (Figure 3), an effect which is unusual in the rodent brain [82]. BPA also reduced *Kiss1* expression in males, an effect which was enhanced in animals maintained on the soy diet, suggesting that this specific effect may be estrogenic. Although adult expression is readily identifiable in the medial amygdala [80], ongoing, concurrent studies in our laboratory indicate that expression is not detectable in pre-weanlings suggesting that a mature HPG axis may be required for maximal expression of *Kiss1*. The functional role of these neurons remains to be delineated. Prior studies have shown that central, but not peripheral, kisspeptin administration elicits oxytocin secretion in females and vasopressin secretion in males, suggesting a role for amygdalar kisspeptin neurons in the modulation of affective behavior [83]. Further anatomical studies are needed to more comprehensively characterize the ontogeny of this neuronal population, their sensitivity to endocrine disruption, and their functional role in sociosexual behaviors.

The hypothesis that early life exposure to EDCs such as BPA and soy phytoestrogens can alter behavior is further supported by parallel work in rodents and humans showing that early life epigenetic changes within the hypothalamic-pituitary-adrenal (HPA) axis, arising from environmental influences, can permanently alter stress sensitivity and coping strategies [84], [85]. It also implicates an epigenetic mechanism of action. Here, EE exposure did not completely recapitulate the behavioral or transcriptional effects of BPA, soy, or the combination of the two, demonstrating that BPA and soy are not simply acting as estrogens. Prior work has established that BPA can induce DNA methylation changes that are blocked by concurrent administration of the soy isoflavone GEN [16], [86], raising the possibility that a similar interaction may have occurred here, resulting in the dietary-dependent behavioral outcomes. One possibility is that differential methylation of the ERβ promoter resulted in decreased expression, and subsequently the decreased expression of *Mc4r*, *Mc3r*, *Gal* and other genes regulated by *Esr2* activity, but more extensive work is needed to test this hypothesis. Understanding the specific cellular and molecular mechanisms by which early life BPA exposure alters behavior is critical for determining if effects observed in animal models have implications for human health.

Finally, it is important to highlight that the dosing method used here produced serum BPA levels at all phases of the project that were equivalent to, or below, those reported in humans [26], [48]. Because BPA was administered in drinking water, exposure was likely low but continuous throughout the day, a pattern that more closely models that of humans than gavage or other methods of bolus administration. Although trace levels of free BPA were observed in some unexposed controls, suggesting an alternative and uncontrolled source of exposure, it may be an artifact of the analysis because the levels were so close to the limit of detection. The most likely source is diet, as we routinely monitor our caging leachate and water to ensure that they are BPA-free. Soy phytoestrogen exposure was monitored by assessing serum GEN and its metabolites. Serum levels were well below those seen in infants exclusively fed soy-based infant formula [87]. It has long been hypothesized that GEN, BPA and other EDCs are not readily metabolized in neonates, and the absence of the glucoronidated form in PND 12 serum is consistent with this view. Serum levels reported here are high enough to induce physiological effects in rat models [26]. Exposure to BPA and GEN was likely lowest during lactation because, although placental transfer of both compounds have been established, neither is known to lactationally transfer readily [26], [49], [88].

### Conclusions

Affective disorders in adults and children have well recognized sex differences in etiology. Boys are at higher risk of autism spectrum disorders, ADHD, and early onset schizophrenia [89], [90] while women disproportionately suffer from anxiety, major depression, panic and eating disorders [91]. Notably, male-biased disorders appear to have their origins in development while female-biased disorders are generally post-pubertal in onset implying that the windows of sensitivity to environmental exposure may be sexually dimorphic with males being more sensitive during development and females later in life [92], [93]. The data shown here reveal the potential for BPA and other EDCs to alter behaviors associated with anxiety, but also the potential for those alterations to be modified/mitigated by a soy-rich diet. These observations are a critical reminder that not all EDCs are synthetic and that plant compounds also have the potential to interact with hormone-sensitive mammalian systems, including the brain. Gene expression data from the amygdala imply a role for Esr2, melanocortins, and oxytocin/vasopressin signaling pathways in the manifestation of these behavioral changes, but future work will be needed to confirm this assertion. Collectively, these data highlight the plasticity of complex behaviors, their sex differences, and their potential for alteration by chemicals in the environment.

Finally, while humans and rodents perceive and express stress and anxiety differently, important core elements of the genetic and neurobiological basis of anxiety phenotypes are evolutionarily conserved across species, particularly in the amygdala and hypothalamus [31], [94], [95], [96], [97], [98]. Thus the rat is an appropriate animal model for understanding how the interaction of BPA and diet influence sociosexual behaviors, and identifying the neural mechanisms by which these changes are induced.

## Supporting Information

Figure S1 **Juvenile elevated plus maze (EPM) closed arm activity is indicative of overall locomotor behavior.** Because no overall effect of gender was identified the data were collapsed across sex for analysis. (A) Two-way ANOVA revealed a main effect of BPA exposure on the number of closed arm entries (*P*≤0.03) indicating that these animals were less active compared to unexposed controls. Post-hoc analysis did not identify significant differences between BPA-exposed animals and controls maintained on the same deit. EE had no impact on this behavior. (B) Soy fed animals spent slightly less time in the closed arms than animals maintained on the soy-free diet. EE had no effect on this behavior. (Graphs depict mean ± SEM).
(DOCX)

Figure S2 **Adult EPM closed arm activity.** (A) Three-way ANOVA revealed a significant sex by diet by BPA interaction (*P*≤0.05) on the number of closed arm entries. Post hoc t-tests within sex identified no sex difference in the number of closed arm entries made by the control animals on the soy-free diet. In all other groups, however, females made significantly more closed arm entries than males (*P*≤0.05). This difference was attributable to the combination of elevated activity in the females, and decreased activity in the males. (B) Time spent in the closed arms was sexually dimorphic in all groups except the BPA soy-free diet group. (Graphs depict mean ± SEM, *P≤0.05, **P≤0.001).
(DOCX)

Table S1 Free levels of BPA and GEN as well as the glucuronidated form of GEN (GEN-gluc) were assessed. BPA levels were within the human range and near the limit of detection. GEN levels were higher in the dams than the pups, reflecting poor lacational transfer. ND = Not detectable.
(DOCX)

Table S2 The original p-value is listed for each gene obtained by parametric (3-way ANOVA) or non-parametric statistics. For parametric tests, main effects and interactions were analyzed. For non-parametric tests data were collapsed across independent variables and analyzed by a Mann-Whine U or Kruskal-Wallis test to determine p-values for combinations of independent variables. Bold text indicates effects of BPA, both main effects and interactions/combinations (p<0.05). One gene, DNA methyltransferase 3l (Dnmt3l) did not amplify well or was not expressed in the amygdala and is not included in the results.
(DOCX)

Supporting Information S1 **Supporting methods.**
(DOCX)
