# Supplementary figures and images for: Anxiogenic Effects of Developmental Bisphenol A Exposure Are Associated with Gene Expression Changes in the Juvenile Rat Amygdala and Mitigated by Soy
Source: PLoS One. 2012 Sep 5;7(9):e43890. doi: 10.1371/journal.pone.0043890 (PMC3434201; doi:10.1371/journal.pone.0043890)

**
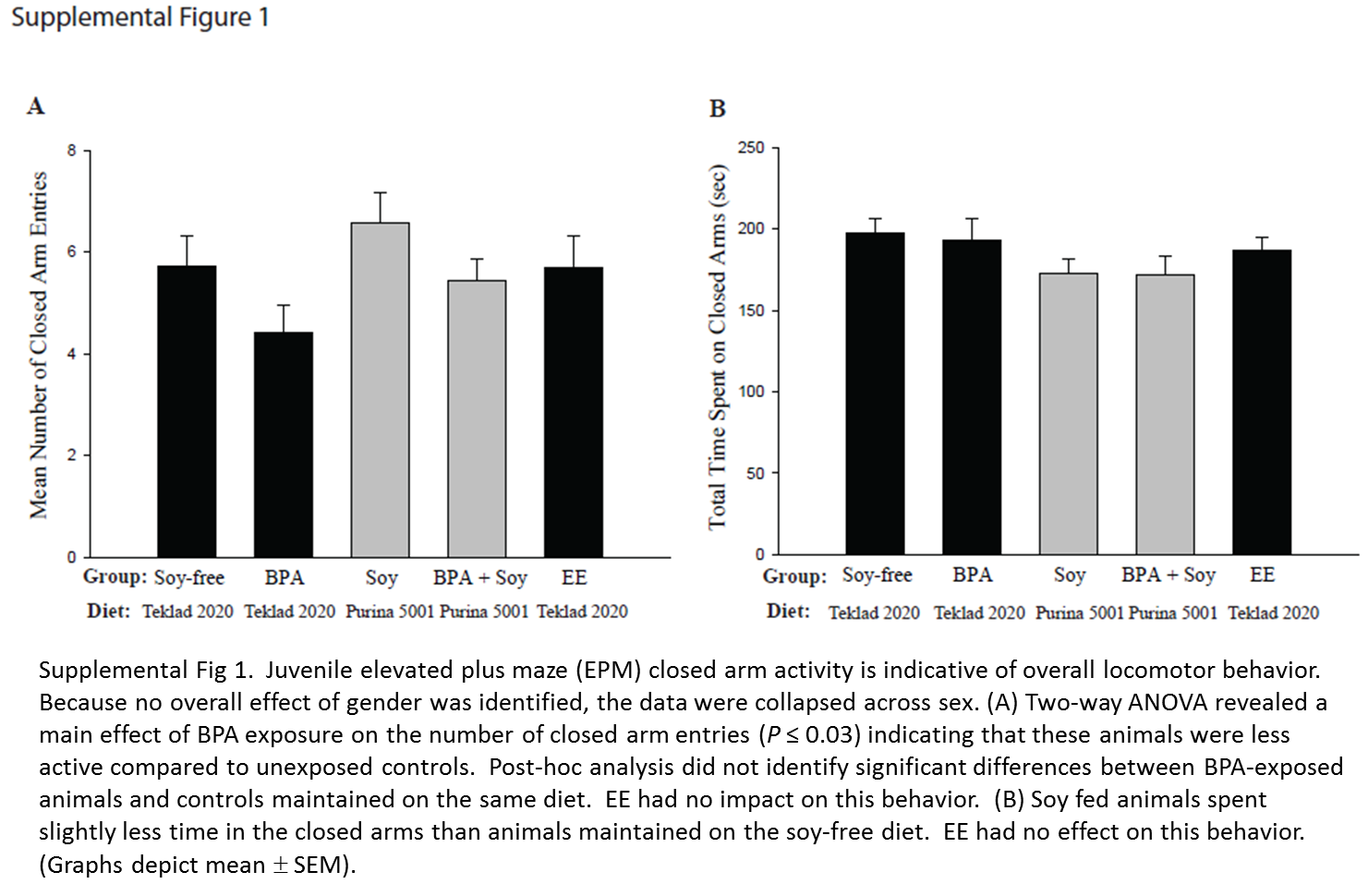
**Figure S1. Juvenile Closed Arm Activity

Supplement: Figure S1 — Juvenile elevated plus maze (EPM) closed arm activity is indicative of overall locomotor behavior. Because no overall effect of gender was identified the data were collapsed across sex for analysis. (A) Two-way ANOVA revealed a main effect of BPA exposure on the number of closed arm entries (P≤0.03) indicating that these animals were less active compared to unexposed controls. Post-hoc analysis did not identify significant differences between BPA-exposed animals and controls maintained on the same deit. EE had no impact on this behavior. (B) Soy fed animals spent slightly less time in the closed arms than animals maintained on the soy-free diet. EE had no effect on this behavior. (Graphs depict mean ± SEM). (DOCX) [file pone.0043890.s001.docx]

Figure S2. Adult Closed Arm Activity


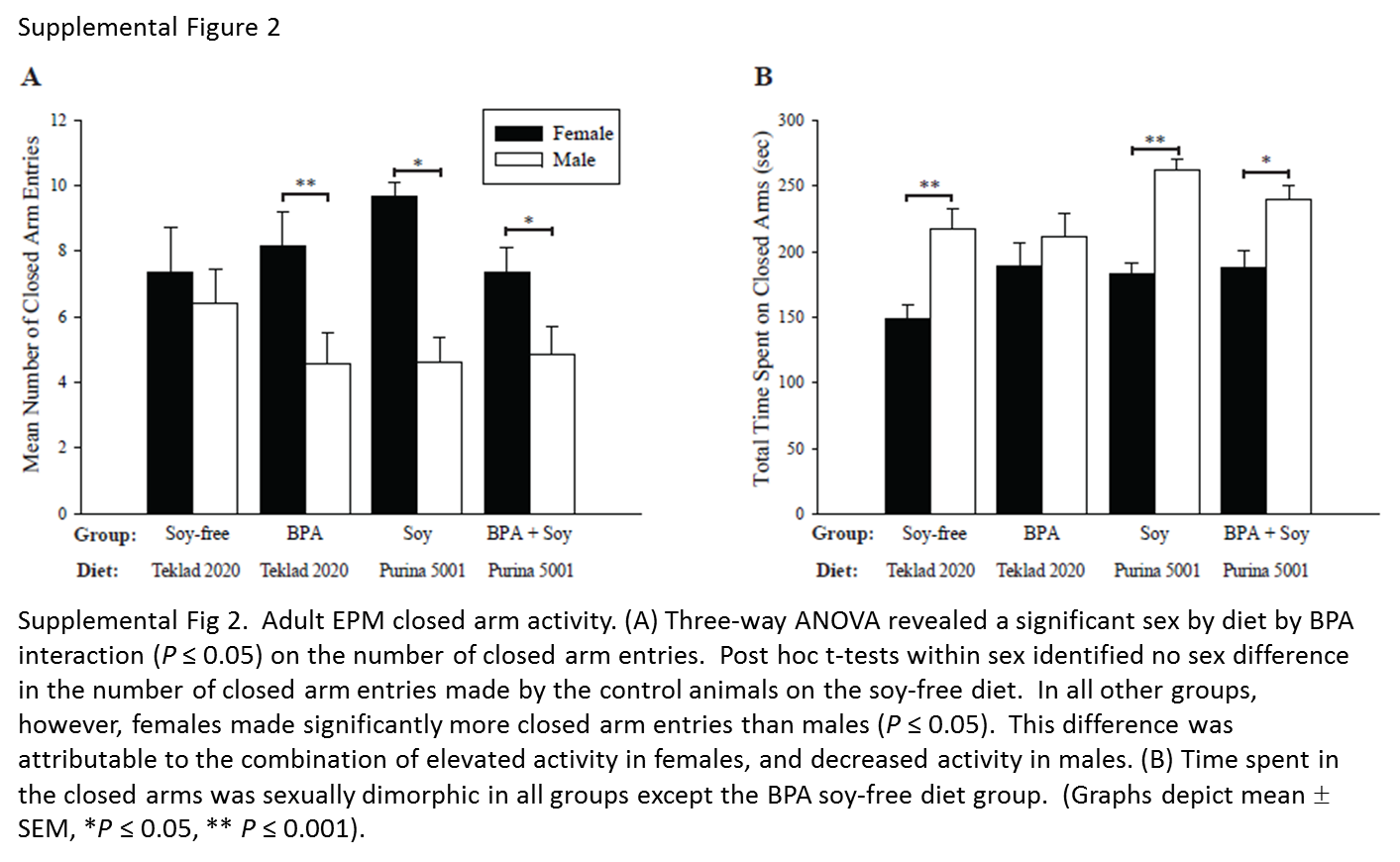

Supplement: Figure S2 — Adult EPM closed arm activity. (A) Three-way ANOVA revealed a significant sex by diet by BPA interaction (P≤0.05) on the number of closed arm entries. Post hoc t-tests within sex identified no sex difference in the number of closed arm entries made by the control animals on the soy-free diet. In all other groups, however, females made significantly more closed arm entries than males (P≤0.05). This difference was attributable to the combination of elevated activity in the females, and decreased activity in the males. (B) Time spent in the closed arms was sexually dimorphic in all groups except the BPA soy-free diet group. (Graphs depict mean ± SEM, *P≤0.05, **P≤0.001). (DOCX) [file pone.0043890.s002.docx]
