# Supplementary material for: Anxiogenic Effects of Developmental Bisphenol A Exposure Are Associated with Gene Expression Changes in the Juvenile Rat Amygdala and Mitigated by Soy
Source: PLoS One. 2012 Sep 5;7(9):e43890. doi: 10.1371/journal.pone.0043890 (PMC3434201; doi:10.1371/journal.pone.0043890)
Supplement: Table S1 — Free levels of BPA and GEN as well as the glucuronidated form of GEN (GEN-gluc) were assessed. BPA levels were within the human range and near the limit of detection. GEN levels were higher in the dams than the pups, reflecting poor lacational transfer. ND = Not detectable. (DOCX) [file pone.0043890.s003.docx]

**Table S1. Mean EDC blood plasma levels (ng/ml) spanning gestation through PND 12.**

**A**

| Dam Exposure Group | BPA | GEN | GEN-gluc |
| --- | --- | --- | --- |
| Soy-free | 0.67 | ND | ND |
| Soy | ND | 20.57 | 0.76 |
| BPA | 0.84 | ND | ND |
| BPA + Soy | 1.24 | 41.01 | 46.41 |

**B**

| PND 12 Exposure Group | Sex | BPA | GEN | GEN-gluc |
| --- | --- | --- | --- | --- |
| Soy-free | Female | ND | ND | ND |
|  | Male | ND | ND | ND |
| Soy | Female | ND | 0.99 | ND |
|  | Male | ND | 1.44 | ND |
| BPA | Female | 0.34 | ND | ND |
|  | Male | 0.20 | ND | ND |
| BPA + Soy | Female | 1.23 | 0.51 | ND |
|  | Male | 0.54 | 0.60 | ND |
