# Supplementary material for: Anxiogenic Effects of Developmental Bisphenol A Exposure Are Associated with Gene Expression Changes in the Juvenile Rat Amygdala and Mitigated by Soy
Source: PLoS One. 2012 Sep 5;7(9):e43890. doi: 10.1371/journal.pone.0043890 (PMC3434201; doi:10.1371/journal.pone.0043890)
Supplement: Table S2 — The original p-value is listed for each gene obtained by parametric (3-way ANOVA) or non-parametric statistics. For parametric tests, main effects and interactions were analyzed. For non-parametric tests data were collapsed across independent variables and analyzed by a Mann-Whine U or Kruskal-Wallis test to determine p-values for combinations of independent variables. Bold text indicates effects of BPA, both main effects and interactions/combinations (p<0.05). One gene, DNA methyltransferase 3l (Dnmt3l) did not amplify well or was not expressed in the amygdala and is not included in the results. (DOCX) [file pone.0043890.s004.docx]

**
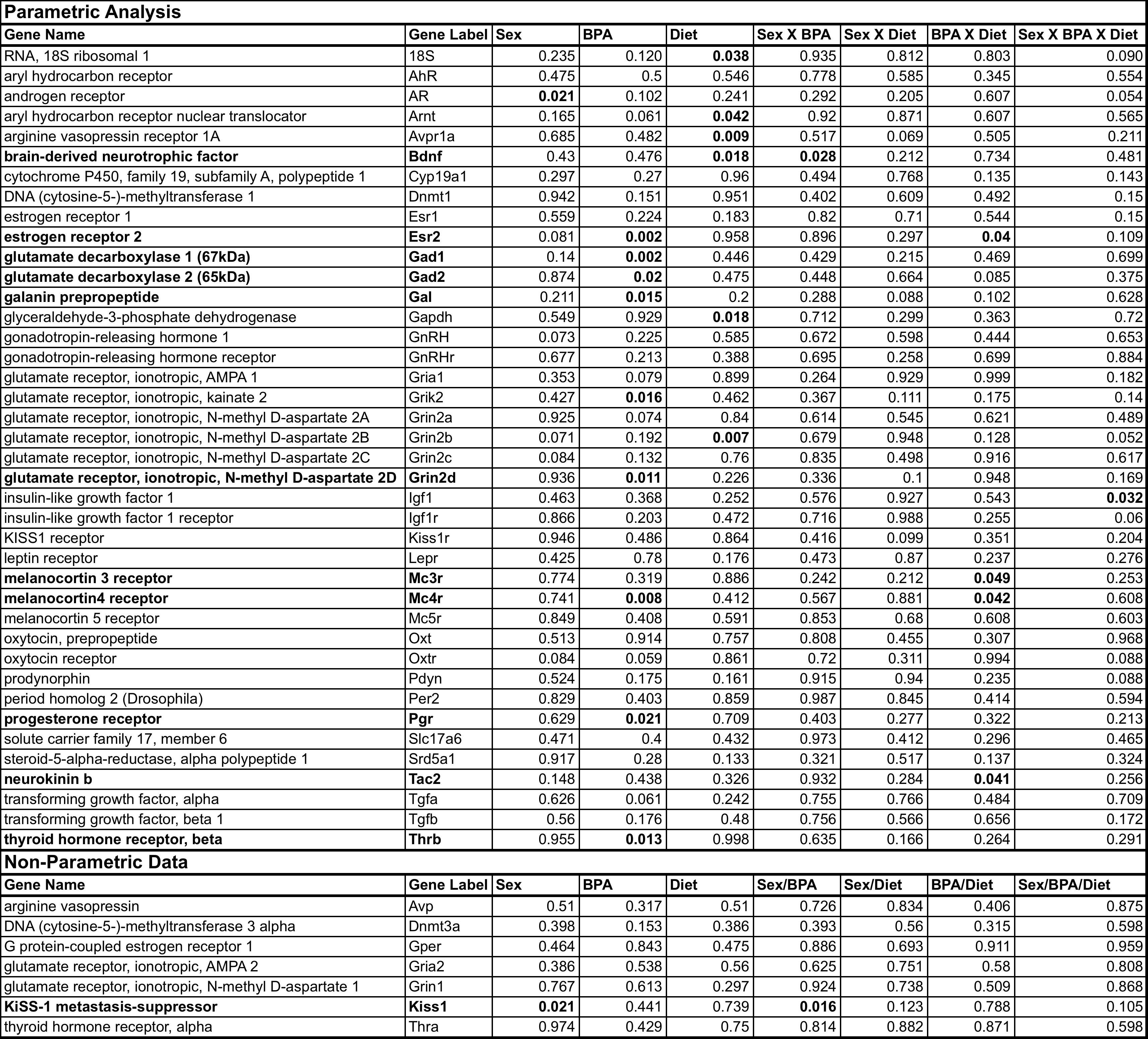
Table S2: List of the 48 genes measured by real-time PCR in the amygdala of male and female rats exposed to BPA, soy diet or BPA and a soy diet**
