# Supplementary material for: Anxiogenic Effects of Developmental Bisphenol A Exposure Are Associated with Gene Expression Changes in the Juvenile Rat Amygdala and Mitigated by Soy
Source: PLoS One. 2012 Sep 5;7(9):e43890. doi: 10.1371/journal.pone.0043890 (PMC3434201; doi:10.1371/journal.pone.0043890)
Supplement: Supporting Information S1 — Supporting methods. (DOCX) [file pone.0043890.s005.docx]

**SUPPORTING INFORMATION S1**

**SUPPORTING METHODS**

**Serum Analysis of BPA and GEN.** Chemicals and supplies were purchased commercially as indicated: bisphenol A (BPA) and ammonium acetate (Sigma-Aldrich, St. Louis, MO, USA); genistein (Indofine Chemical, Hillsborough, NJ, USA); bisphenol A β-D-glucuronide and genistein 4’-β-D-glucuronide (TRC Canada, North York, Ontario); pesticide grade ethyl acetate, liquid chromatography/mass spectrometry grade methanol, and formic acid (Thermo Fisher Scientific, Waltham, MA, USA; high purity and high performance liquid chromatography grade solvents acetonitrile and water (Honeywell Burdick & Jackson, Muskegon, MI, USA; and Oasis HLB (Hydrophilic Lipophilic Balance) solid phase extraction (SPE) cartridges (30 mg, 1 mL; Waters, Milford, MA USA).

*Sample Preparation:* Samples consisted of 100 μL rat plasma, 80 μL of 1 M formic acid, 100 μL of 250 mM ammonium acetate (pH 5) and water to achieve a total volume of 1.0 mL. After 5 minutes of sonication and 10 minutes of centrifugation at 2,500 rpm, solid phase extraction was performed to separate BPA, GEN, and GEN 4’-β-D-glucuronide from their matrix. Oasis HLB SPE cartridges were conditioned using 3 mL methanol and 3 mL water prior to loading the sample. SPE cartridges were washed with 2 mL of 9:1 water/methanol (*v/v*). Analytes of interest were eluted using sequential additions of 3 mL ethyl acetate, 3 mL methanol, and 3 mL acetonitrile at a flow rate of approximately 2 mL/min. Eluate was evaporated to dryness under a stream of nitrogen and reconstituted in 50% acetonitrile in water for direct injection into HPLC/ITMS.

*Standard Solutions:* Analytes were initially dissolved in methanol (GEN) or acetonitrile (BPA, GEN 4’-β-D-glucuronide). All subsequent standard solutions were prepared via serial dilution in acetonitrile and stored at -20°C for up to three months.

*Controls:* Solid phase extraction controls were run alongside each batch of samples tested. Blank solid phase extraction controls contained high purity water instead of rat plasma and positive solid phase extraction controls contained high purity water spiked with BPA, GEN, and GEN 4’-β-D-glucuronide.

*Analysis Conditions:* The optimized analytical conditions identified previously in our lab [[1](#_ENREF_1)]were used, with minor modifications. A Discovery C8 high performance liquid chromatography column (50 x 4.6 mm I.D., 5 μm; Supelco, St. Louis, MO, USA), and a KrudKatcher Ultra In-Line Filter guard column (0.5 μm; Phenomenex, Torrence, CA, USA) were used for chromatographic separation. Mobile phase A was 10% acetonitrile in 2 mM ammonium acetate (pH 9), and mobile phase B was acetonitrile. The gradient used was: 0 to 3 min 45% B, 3 to 14 min 45 to 89% B, 14 to 18 min 89% B, 18 to 18.2 min 89 to 45% B, and 18.2 to 20 min 45% B. Analytes were identified and quantified on a ThermoFinnigan LTQ mass spectrometer (Thermo Fisher Scientific), using electrospray ionization (ESI) in negative mode, and a linear ion trap as an analyzer, all under regulation of Xcalibur 2.0.7 software. LC/MS experiments were used for quantitation of BPA and GEN while LC/MS^3^was used for quantitation of GEN glucuronide. The MS/MS transitions were previously described, while the MS^3^ transitions for Gen glu were as follows: 445 m/z to 269 m/z to 197 m/z .

**Gene Expression Analysis.** A subset of animals tested for juvenile behavior was sacrificed on PND 34, and the brains removed and flash frozen on powdered dry ice. Each brain was cryosectioned to the caudal border of the amygdala (identified by local landmarks with the assistance of a brain atlas (Paxinos and Watson plates 49-58)). The amygadala was removed via micropunch, and shipped to Dr. Gore at University of Texas Austin for further analysis according to their established protocols [[2](#_ENREF_2),[3](#_ENREF_3)]. RNA was isolated using the All-Prep**™** DNA RNA Micro Kit (Qiagen, Valencia, CA, USA) according to the manufacturer’s protocol. After elution, samples were treated with 1U TURBO DNase (Ambion, Foster City, CA, USA), concentrated via ethanol precipitation and resuspended in nuclease–free water. Samples were run on a Bioanalyzer 2100 (Agilent, Cedar Creek, TX, USA) to assess RNA purity, integrity, and concentration and 500ng was converted to cDNA using a High Capacity RT Kit (Applied Biosystems, Foster City, CA, USA).The Taqman**®** low-density PCR array (Applied Biosystems Inc, Foster City, CA, USA) was used to quantify expression of 48 genes in individual amygdalar dissections [[3](#_ENREF_3)] using the ViiA7 real-time PCR machine (Applied Biosystems Inc, Foster City, CA, USA) and Taqman**®** real-time PCR reagent (Applied BiosystemsInc, Foster City, CA, USA). Relative expression was determined using the comparative Ct method [[4](#_ENREF_4),[5](#_ENREF_5),[6](#_ENREF_6)], with each sample normalized to *Gapdh*, and data calibrated to the median **Δ**Ct for the group with the lowest expression (set at 1.0 [[3](#_ENREF_3)]) to determine fold change in expression for each sample.

**REFERENCES**

1. Coughlin JL, Winnik B, Buckley B (2011) Measurement of bisphenol A, bisphenol A ss-D-glucuronide, genistein, and genistein 4'-ss-D-glucuronide via SPE and HPLC-MS/MS. Analytical and bioanalytical chemistry 401: 995-1002.

2. Jakubowski M, Blum M, Roberts JL (1991) Postnatal development of gonadotropin-releasing hormone and cyclophilin gene expression in the female and male rat brain. Endocrinology 128: 2702-2708.

3. Walker DM, Juenger TE, Gore AC (2009) Developmental profiles of neuroendocrine gene expression in the preoptic area of male rats. Endocrinology 150: 2308-2316.

4. Pfaffl MW (2001) A new mathematical model for relative quantification in real-time RT-PCR. Nucleic Acids Res 29: e45.

5. Livak KJ, Schmittgen TD (2001) Analysis of relative gene expression data using real-time quantitative PCR and the 2(-Delta Delta C(T)) Method. Methods 25: 402-408.

6. Schmittgen TD, Livak KJ (2008) Analyzing real-time PCR data by the comparative C(T) method. Nat Protoc 3: 1101-1108.
